# Organ-sparing surgery of penile cancer: higher rate of local recurrence yet no impact on overall survival

**DOI:** 10.1007/s00345-019-02793-9

**Published:** 2019-05-06

**Authors:** Andrea Katharina Lindner, Gert Schachtner, Eberhard Steiner, Alexander Kroiss, Christian Uprimny, Fabian Steinkohl, Wolfgang Horninger, Isabel Heidegger, Stephan Madersbacher, Renate Pichler

**Affiliations:** 1 Department of Urology, Medical University Innsbruck, Anichstraße 35, 6020 Innsbruck, Austria; 2 Department of Nuclear Medicine, Medical University Innsbruck, Innsbruck, Austria; 3 Department of Radiology, Medical University Innsbruck, Innsbruck, Austria; 4 Department of Urology, Kaiser Franz Josef Hospital, Sigmund Freud Private University, Vienna, Austria

**Keywords:** Penile neoplasm, Squamous cell, Recurrence, Organ-preserving surgery, Survival, Follow-up

## Abstract

**Purpose:**

To report on the oncological outcome of organ-sparing surgery (OSS) compared to (total or partial) penectomy regarding recurrence patterns and survival in squamous cell carcinoma (SCC) of the penis.

**Methods:**

This was a retrospective study of all patients with penile SCC and eligible follow-up data of at least 2 years at our institution. Patients with tumors staged ≥ pT1G2 underwent invasive lymph node (LN) staging by dynamic sentinel-node biopsy or modified inguinal lymphadenectomy. Radical inguinal lymphadenectomy was performed when LNs were palpable at diagnosis and in those with a positive LN status after invasive nodal staging. Follow-up visits were assessed, and local, regional and distant recurrences were defined and analyzed.

**Results:**

55 patients were identified with a mean follow-up of 63.7 months. Surgical management was OSS in 26 patients (47.2%) and partial or total penectomy in 29 cases (52.8%). Histopathological staging was: pTis (12.7%), pTa (16.3%), pT1a (18.2%), pT1b (5.5%), pT2 (29.1%) and pT3 (18.2%), respectively. Patients in the penectomy group were significantly older (mean 68 vs. 62 years; *p* = 0.026) with a higher rate of advanced tumor stage (≥ pT2: 44.8% vs. 11.5%; *p* = 0.002). The local recurrence rate was 42.3% (*n* = 11) following OSS compared to 10.3% (*n* = 3) after penectomy (*p* = 0.007). Kaplan–Meier curves showed no significant differences between the two groups regarding metastasis-free and overall survival.

**Conclusions:**

OSS is associated with a higher local recurrence rate compared to penectomy, yet it has no negative impact on overall and metastasis-free survival.

**Electronic supplementary material:**

The online version of this article (10.1007/s00345-019-02793-9) contains supplementary material, which is available to authorized users.

## Introduction

Penile cancer is a rare tumor accounting for 0.4–0.6% of malignant diagnoses in Europe and the USA with a higher incidence in developing countries [1] with the highest global age-standardized incidence in the state of Maranhão in Brazil (6.15 per 100.000) [2]—occurring at a mean age of 60–70 years [3]. The primary site of SCC is the glans penis in 48% of diagnosed cases; followed by 21% affecting the prepuce, 9% involving both glans penis and prepuce, 6% emerging from the coronal sulcus and 2% the shaft [4]. The most common histopathological tumor type is squamous cell carcinoma (SCC), followed by warty, papillary and basaloid carcinoma [5]. Risk factors for development of penis carcinoma are phimosis [6] with concomitant repeated balanitis [7], poor hygiene [8] as well as the presence of lesions sporadically associated with SCC of the penis such as balanitis xerotica obliterans [9]. Only limited data on the oncological outcome after surgical intervention for penile cancer are available, due to its rare incidence. Guideline recommendations are currently based on retrospective reports published by supra-regional referral centers [10, 11] and have only changed little over the past years. Radical partial or total penectomy is still associated with significant functional, sexual and psychological deficits, despite high oncological control rates. “Organ-preserving” techniques such as laser therapies with Nd:YAG or CO_2_ laser [12, 13], partial or total glansectomy with reconstruction, and wide local excision with intra-operative frozen sections have been shown to maintain penile form and function without reducing oncological long-term control [14]. In the past, organ-preserving surgical methods have become increasingly popular to treat localized penile cancer [10], as they improve functional outcome such as sexual and urinary functions, quality of life, body image and well-being [15, 16].

The aim of this retrospective observational study was to evaluate long-term oncological outcomes following organ-sparing surgery compared to radical surgery such as penile amputation (total or partial) and to analyze recurrence patterns and their impact on survival to substantiate the current trend to penile-preserving cancer surgery.

## Patients and methods

This is an observational study based on a retrospective analysis of the uro-oncology cancer database of the Department of Urology, Medical University Innsbruck (study number 1006/2017). Research work was performed in accordance with the 1964 Helsinki declaration, its later amendments and institutional ethical standards based on good clinical practice [17].

### Patients and pathological staging

**Fig. 1:**
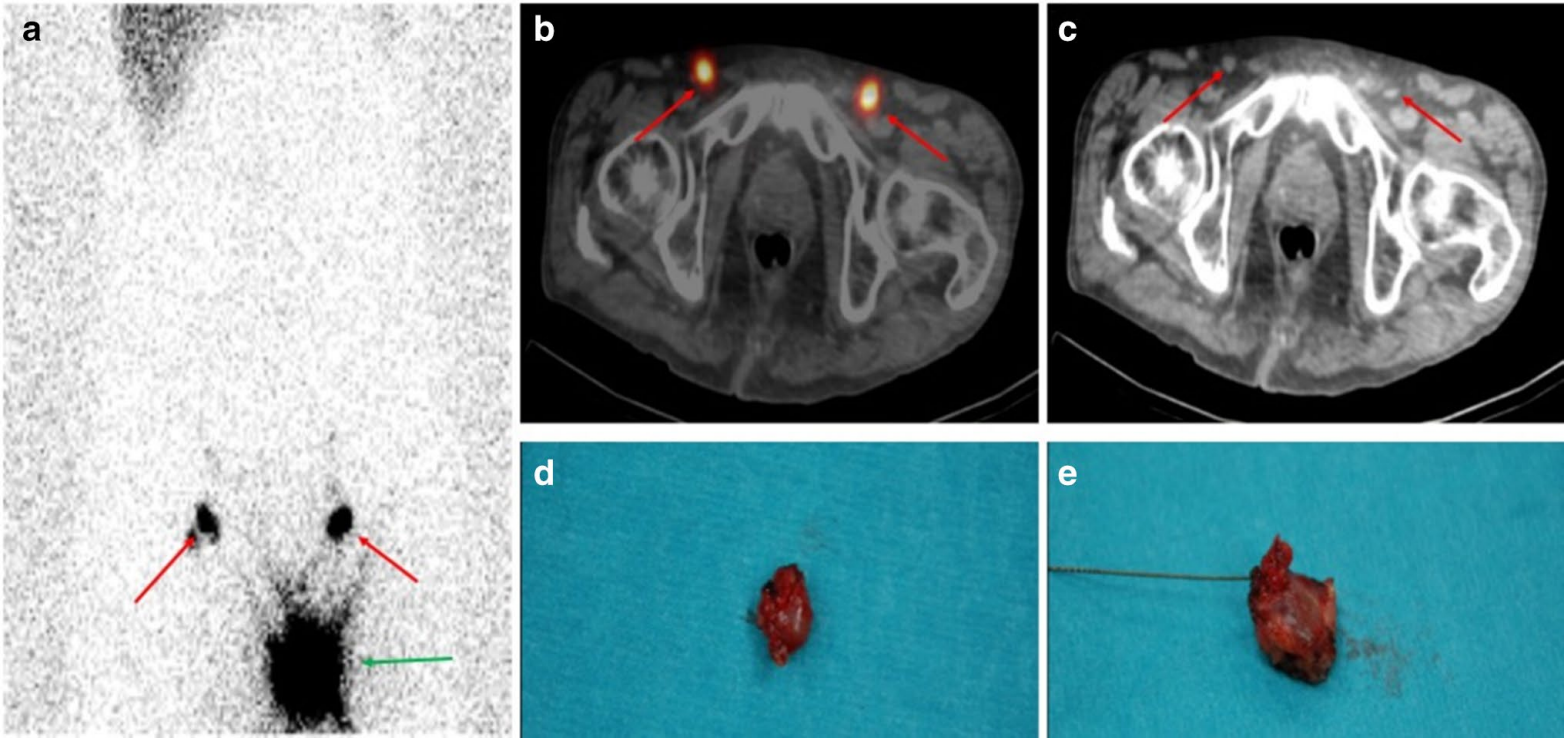
Sentinel lymph node biopsy (SLNB) guided by lymphoscintigraphy [39]. In addition to early dynamic imaging (not displayed) following intradermal injection of 40 MBq Technetium-99 m-labelled nanocolloidal albumin (99mTc-Nanocoll^®^) peritumorally, planar static imaging 60 min post-injection of the pelvis is performed, including combined single-photon-emission tomography with low-dose computed tomography (SPECT/CT) acquisition. On planar image 60 min p.i. apart from the injection site (**a** green arrow), intense focal tracer uptake is visualized in the right and left pelvic area (**a** red arrows). On fused SPECT/CT images (**b** axial slice, red arrows), focal uptake is located in the inguinal region, that corresponded to non-enlarged lymph nodes on low-dose CT (**c** axial slice, red arrows), representing inguinal SLN. Both SLN were localized intra-operatively with a gamma-probe 1 day after tracer injection and could be surgically removed (**d**, **e**). Final histology confirmed pN0

Medical records of all penile cancer patients diagnosed between 1971 and 2016, and who underwent penile tumor resection (radical or organ-preserving) were reviewed retrospectively. All patients with a confirmed histopathology of SCC and eligible follow-up data of at least 2 years at our oncology outpatient department were included. Detailed patient characteristics, tumor treatment and follow-up data were evaluated; patients with benign findings or no SCC on definite histopathology, those who were seen elsewhere post-operatively, or who had evidence of distant or local metastasis on imaging before surgery were excluded. Staging was performed according to the 2016 tumor-node-metastasis (TNM) classification [18]. Grade groups were defined grades 1–4 according to the amount of undifferentiated cells, based on Broder’s histopathological scheme [19]. Invasive lymph node staging (pN) was recommended for patients with no palpable inguinal nodes (cN0), but pT1 tumors of intermediate and high risk (≥ pT1G2), as well as for T2–T4 tumors by either dynamic sentinel-node biopsy (DSNB) (Fig. 1) or by modified inguinal lymphadenectomy (miLAD), [5, 20]. Radical inguinal lymphadenectomy (rLAD) was performed in patients with palpable inguinal lymph nodes (cN1/N2) at primary diagnosis, and in those with a positive lymph node status after invasive nodal staging [5].

### Primary tumor treatment

“Organ-sparing” surgery (OSS) included laser treatment, local excision, partial or total glansectomy with reconstruction or resurfacing. Intra-operative frozen sections were performed to assess surgical margins. In case of R1 or R2 resection, repeated wide local excision was applied to achieve a minimum of 3–4-mm negative surgical margins [21]. Tumors staged with pTis, pTa and pT1 were mainly treated with OSS. Three pT2 tumors which were localized distally were also managed with primary organ-sparing therapy.

In contrast, “radical” surgery was defined as partial penectomy or total penectomy with perineal urethrostomy. Almost all patients with proximally located pT2 tumors and pT3 tumors (with invasion of the urethra) underwent partial or total penile amputation.

### Recurrence

Recurrence was defined as reappearance after primary surgical tumor treatment: local, regional or distant. Recurrence of the primary tumor to the penis was described as “*local recurrence*”, inguinal and/or pelvic lymph node metastasis was described as *“regional recurrence*” and “*distant recurrence*” in the case of distant lymphatic or hematogenous metastases.

Local recurrence was treated with either a second OSS if there was no corpus cavernosum invasion or partial/total amputation when a large or high-stage recurrence was diagnosed. Regional recurrence was mainly treated with a multimodal treatment concept including radical inguinal lymphadenectomy and neoadjuvant and/or adjuvant chemotherapy depending on definitive histopathology, primary non-resectable or fixed inguinal lymph nodes. Palliative chemotherapy was offered to patients with systemic distant disease.

### Follow-up

According to our institutional practice, follow-up visits were scheduled every 3 months in the first three post-operative years, then at 6-month intervals until the end of the 5th year, and once yearly thereafter. Each control included assessment about patient`s sexual and urinary quality of life, complete laboratory blood examination, urinary dipstick analysis with measurement of residual urine, physical inguinal and penile examination with photo documentation. Imaging (chest and abdominopelvic CT scan) was performed at every third control, alternated with chest radiography, abdominal and inguinal ultrasound.

### Statistical analyses

Patient and tumor characteristics of the applied penile surgery method (organ-preserving versus radical surgery) were compared by the Mann–Whitney *U* test and Pearson chi-square test for nominal parameters. Overall survival (OS), local recurrence-free survival and metastasis-free survival were defined as the time period from the date of primary tumor diagnosis to death of any cause, and the detection of local penile recurrence and distant or regional lymphatic metastases. The data recorded at the date of last control were used as the endpoint of those patients who were still on routine follow-up at the end of our study. Survival analysis was performed with Kaplan–Meier survival curves; these were compared using the log-rank test. Statistical analyses were performed using SPSS (v24, IBM Corp., Armonk, NY) with two-sided *p* < 0.05 considered as statistically significant. Kaplan–Meier plots were produced with GraphPad PrismTM6 (GraphPad Software Inc., La Jolla, CA).

## Results

### Patient characteristics

55 patients with a mean (± SD) age of 65.2 ± 1.8 (median: 66; range, 26–90) years who underwent surgical resection for penile SCC were included. The mean (± SD) follow-up was 63.7 (± 11.9) months. Histopathological staging of penile lesions showed pTis (12.7%), pTa (16.3%), pT1a (18.2%), pT1b (5.5%), pT2 (29.1%) and pT3 (18.2%), respectively. The most frequent tumor grade was grade 2 in 32 cases (58.2%), grade 3 in 12 (21.8%) and grade 1 in 11 (20%) patients. At primary diagnosis, most patients (85.5%; *n* = 47) were not circumcised and 89.1% of penile SCC were located on the glans and/or foreskin. A detailed overview of patient characteristics is presented in Table 1.

**Table 1:** Descriptive patient and histopathological characteristics of the study population (overall and stratified by penile surgical approach), *n* = 55

Patients	Treatment			
	OSS (%)	(Partial/total) amputation (%)	Total (%)	*p* value
*n*	26	29	55	
Age* (years)				
Mean ± SD	61.1 ± 14	68.9 ± 12.2	65.2 ± 1	***p*** = **0.026**
Median	62	68	66	
pT stage**, *n* (%)				***p*** = **0.002**
pTis (%)	7 (26.9%)	–	7 (12.7%)	
pTa (%)	7 (26.9%)	2 (6.9%)	9 (16.3%)	
pT1a (%)	8 (30.8%)	2 (6.9%)	10 (18.2%)	
pT1b (%)	1 (3.8%)	2 (6.9%)	3 (5.5%)	
pT2 (%)	3 (11.5%)	13 (44.8%)	16 (29.1%)	
pT3 (%)	–	10 (35.55)	10 (18.2%)	
Tumor grade**, *n* (%)				*p* = 0.908
Grade 1	7 (26.9%)	4 (13.8%)	11 (20%)	
Grade 2	16 (61.6%)	16 (55.2%)	32 (58.2%)	
Grade 3	3 (11.5%)	9 (31%)	12 (21.8%)	
Clinical lymph node status (cN)**, *n* (%)				
cN0	23 (88.5%)	21 (72.4%)	44 (80%)	*p* = 0.476
cN1/N2	3 (11.5%)	8 (27.6%)	11 (20%)	
cN3	–	–	–	
DSNB/miLND**, *n* (%)	9 (34.65%)	11 (37.9%)	20 (36.7%)	*p* = 0.576
rLAD**, *n* (%)	3 (11.5%)	12 (41.4%)	15 (27.3%)	*p* = 0.929
pN status (DSNB/miLAD)**, *n* (%)				*p* = 0.129
pN0	9 (100%)	7 (63.6%)	16 (29.1%)	
pN1	–	4 (36.4%)	4 (7.2%)	
pN2/pN3	–	–	–	
pN status (rLAD)**, *n* (%)				*p* = 0.774
pN0	2 (75%)	7 (58.3%)	9 (16.4%)	
pN1	1 (25%)	5 (41.7%)	6 (10.9%)	
pN2/pN3	–	–	–	
Local recurrence**, *n* (%)				***p*** = **0.007**
Yes	11 (42.3%)	3 (10.3%)	14 (25.5%)	
No	15 (57.7%)	26 (89.7%)	41 (74.5%)	
Regional recurrence**, *n* (%)				*p* = 0.613
Yes	1 (3.8%)	3 (10.3%)	4 (7.2%)	
No	25 (96.2%)	26 (89.7%)	51 (92.7%)	
Distant metastasis**, *n* (%)				*p* = 0.259
Yes	2 (7.7%)	4 (13.8%)	6 (10.9%)	
No	24 (92.3%)	25 (86.2%)	49 (89.1%)	

Bold values indicate *p* < 0.05 was considered as statistically significant

*OSS* organ-sparing surgery, *DSNB* dynamic sentinel-node biopsy, *miLND* modified inguinal lymph node dissection, *rLAD* radical inguinal lymphadenectomy

*p* values were calculated by Mann–Whitney *U* test* and Pearson chi-square test**

### OSS versus (partial or total) penectomy

The primary tumor management of 26 (47.2%) patients was with OSS, whereas 29 (52.8%) patients underwent partial or total penectomy. Most patients (*n* = 23, 88.5%) who received OSS were staged pTis, pTa and pT1; three patients with tumor staged pT2 also underwent organ-preserving surgery. Laser ablation was performed in four (15.3%) pTis cases, two (7.7%) patients received primary circumcision and 20 (76.9%) underwent wide local excision with negative intra-operative frozen sections, partial or total glansectomy. An overview of pT stage, recurrence rates and detailed OSS approaches is described in Supplementary Figure 1. Of 29 penile amputations, 26 (89.6%) patients received partial penectomy. In three (10.4%) cases, total penectomy with perineal urethrostomy was necessary because of extensive local spread of primary tumor (pT3) and invasion of the urethra. Three further patients underwent total penectomy due to local recurrence after partial penectomy on follow-up.

Table 1 summarizes the descriptive and histopathological patient characteristics according to surgical approach-related differences. Patients who underwent penectomy were significantly older than those undergoing OSS (mean: 68.9 vs. 61.1 years; *p* = 0.026). Moreover, patients of the penectomy group had a significant greater propensity for advanced tumor stage (≥ pT2: 79.3% vs. 11.5%; *p* = 0.002) but lower rate of local recurrence (10.3% vs. 42.3%; *p* = 0.007) compared to patients with OSS, Table 1.

### Inguinal lymph node management

All nine (34.6%) patients managed with invasive nodal staging after OSS were staged as pN0. The 11 (37.9%) patients of the penectomy group who were managed with primary invasive nodal staging had pN0 in seven (63.6%) cases and pN1 in four (36.4%) cases, Table 1. rLAD was performed in only three (11.5%) patients of the OSS group, confirming pN1 in one patient. In contrast, rLAD was necessary in 12 (41.4%) patients of the penectomy group: 4 patients with pN1 during DSNB and 8 patients with palpable inguinal lymph nodes (cN1/N2) at primary diagnosis. Five (41.6%) of these 12 cases had a positive lymph node status. A detailed overview of the performed penile surgical approach, pT stage, cN and pN status after DSNB/miLND or rLAD is shown in Supplementary Figure 2.

### Management of local recurrent disease

Following local recurrence after primary OSS, 11 (42.3%) patients needed second local surgery after a median disease-free interval of 22 months (range 4–357). Depending on tumor invasion and staging, eight patients received further OSS while the remaining three (27.3%) had to undergo consecutive partial penectomy. Four (15.3%) patients were in need for a third local surgical intervention after repeated local recurrence (Supplementary Figure 1). After primary (partial/total) penectomy, only three (10.3%) patients had local recurrence after a median follow-up of 22 months (range 5–66) and needed further penile surgery resulting in total penectomy.

### Survival analyses

**Fig. 2:**
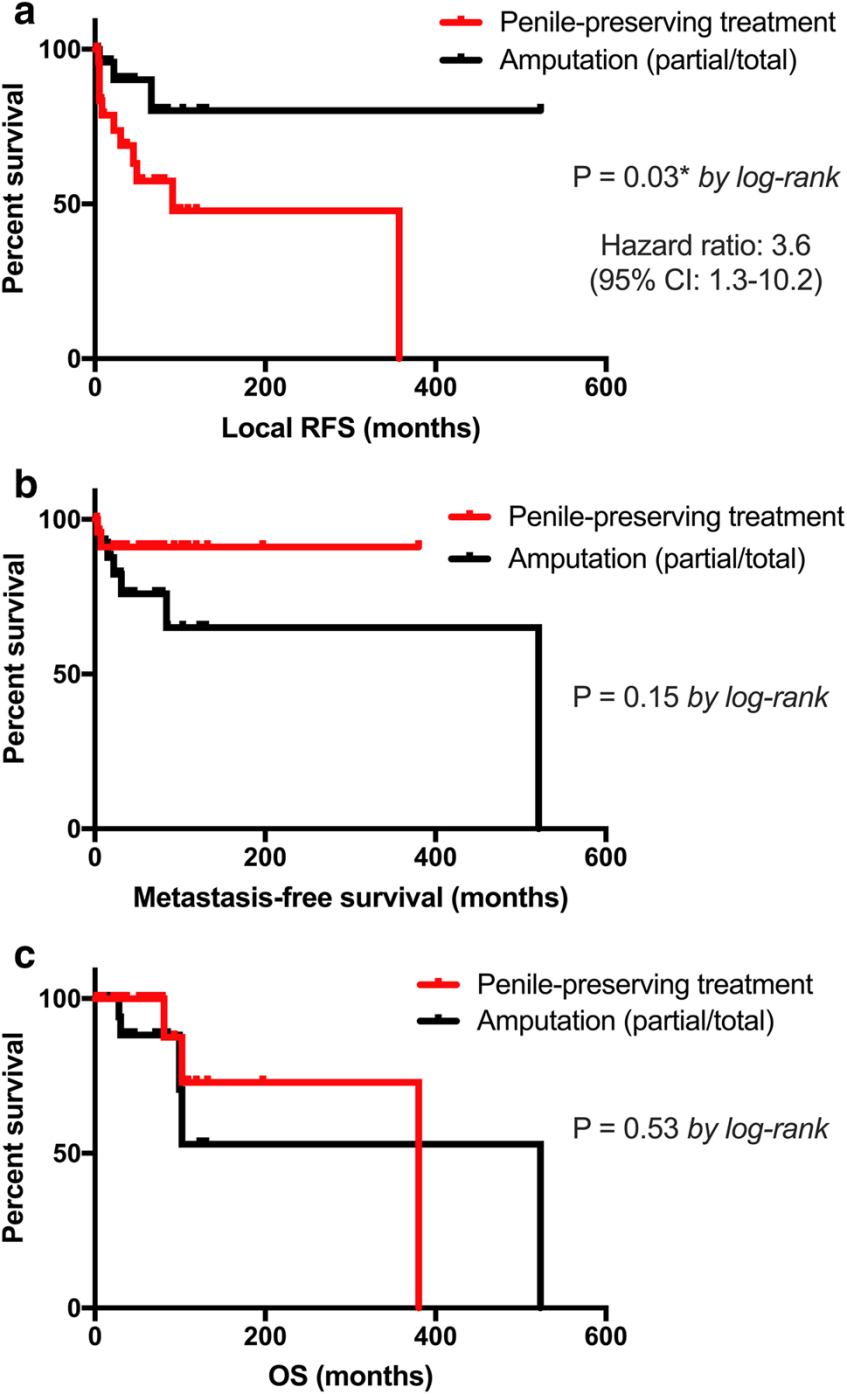
Kaplan–Meier survival curves. **a** Local recurrence-free survival (RFS), **b** (lymphatic and hematogenous) metastasis-free survival and **c** overall survival (OS) in months according to the type of penile surgical approach (OSS vs. penile amputation). *p* values by log-rank test; *p < 0.05; **p < 0.01; ***p < 0.001

The mean local RFS, metastasis-free survival and OS for the entire cohort were 55.1, 62.1 and 63.7 months, respectively. Kaplan–Meier curves comparing the surgical approach (OSS vs. partial/total penectomy) for local RFS, metastasis-free survival and OS are shown in Fig. 2a–c, respectively. Survival analysis revealed a significant negative association between OSS and local RFS (median: 91 months vs. NE; *p* = 0.033) compared to the penectomy group. There were no significant differences between the two groups regarding metastasis-free survival (median OSS vs. penectomy: NE vs. 521 months; *p* = 0.151) and OS (median OSS vs. penectomy: 380 vs. 523 months; *p* = 0.532).

## Discussion

Our data present the oncological outcome and long-term follow-up of a retrospective series of patients receiving penile surgery (OSS versus partial/total penectomy) after primary diagnosis of penile SCC. Partial or total penectomy have both shown to result in good local control with a low risk of local recurrence of 4–5% [22], yet carry the burden of sexual dysfunction and reduced functional and urinary quality of life [23, 24]. Current evidence suggests that OSS maintains a sexual and urinary function, and psychological health [25–27]. Various studies evaluating sexual and urinary dysfunction after penile-preserving surgery using different questionnaires (e.g. International Index of Erectile Function questionnaire; IIEF) [28] confirmed excellent overall urinary function and quality of life [16, 26, 29]. Patients with (partial) penectomy reported more sexual and urinary problems than those treated with OSS [23, 30].

About 80% of carcinomas affect the distal penile region [4] such as the glans and/or foreskin, thus making organ-sparing surgery technically and oncologically feasible. Nevertheless, it is well known that local recurrence is more likely to occur following OSS in up to 42% of patients during the first 5 years, with a high rate (50%) of late recurrence [31], but with no negative impact on long-term survival [10, 21, 32]. A high local recurrence rate in primary preservative penile surgery compared to partial or total penectomy was also reported in a retrospective study by Leijte et al. [22].

Our findings report a local recurrence rate following OSS of 42.3% in comparison with penectomy (10.3%), occurring after a median post-operative follow-up of 22 months. Interestingly, neither metastasis-free survival nor OS were significantly influenced by the extent of penile surgery. Although local recurrence rate was higher after OSS, the majority of patients were managed with further OSS, extended surgery such as partial penectomy only being necessary in 27.3% of patients. Thus, OSS is a safe alternative to radical amputation [14, 26, 27, 33, 34]. Tumors staged pT2 and above were mainly managed by partial penectomy in our study; but there is no general consent concerning recommended techniques in invasive penile cancer [35].

Primary histopathological and nodal staging are the most important prognostic factors in penile cancer as survival rates are known to decrease with advanced staging and with positive nodal spread [36–38]. Our study showed a higher rate of advanced tumor stage in patients who received penectomy, resulting in a notable but not statistically significant trend for a poorer metastasis-free and OS. In contrast to local recurrence patterns, regional recurrence (10.3%) and/or distant metastasis (13.8%) were more frequent in those patients treated with partial or total penectomy, which is most likely due to the fact that patients who underwent (partial) penectomy had a higher primary tumor staging (≥ pT2: 44.8% vs. 11.5%) with increased positive inguinal lymph node status at primary invasive nodal staging (36.4% vs. 0%), thus being at higher risk for distant cancer spread on follow-up. Previous studies have shown that narrow surgical resection margins of only a few millimeters are sufficient to control local disease without affecting OS [39, 40], so that penile-preserving surgery can spare a considerable amount of tissue [21, 22] allowing a paradigm shift towards more organ-sparing approaches. Whichever surgical technique is chosen to manage the primary tumor lesion, lymph node management should be based on the recommended guidelines [5]. Primary DSNB in patients with ≥ pT1G2 without palpable inguinal lymph nodes has matured into a reliable staging method with lower morbidity rates than rLND [5, 38, 39]. To ensure a good long-term oncological outcome with early detection of local recurrence, organ-preserving surgery needs a good patient compliance and should always be followed by careful patient management with regular physician and self-investigation, and regular oncological follow-up visits.

One of the major limitations of this observational study is (i) the limited number of patients with retrospectively evaluated oncological results and (ii) no comprehensive, standardized assessments of post-operative sexual function using the IIEF questionnaire. Our report is demonstrating the efficacy and good long-term oncological outcome of organ-preserving surgery in penile cancer. Prospective studies are needed to corroborate current surgical and therapeutic management principles.

In summary, organ-preserving surgery for penile squamous cell carcinoma is an oncologically safe treatment option with good cosmetic and functional results after careful patient selection based on tumor staging and tumor localization. It is associated with an increased rate of local recurrence, thus requiring both regular self-investigation and frequent follow-up visits to ensure early detection of local recurrence; but it has no negative impact on long-term overall and metastasis-free survival.

## Electronic supplementary material

Below is the link to the electronic supplementary material.

Supplementary Figure 1. Schematic overview of OSS approach, pT stage and local recurrence rates (TIFF 7658 kb)

Supplementary Figure 2. Schematic overview of inguinal lymph node management stratified by penile surgical approach (OSS vs. partial/total penectomy) (TIFF 7247 kb)

## Abbreviations

cN: Clinical inguinal lymph node status
DSNB: Dynamic sentinel lymph node biopsy
IIEF: International index of erectile function
LND: Lymph node dissection
miLAD: Modified inguinal lymphadenectomy
NE: Not estimated
OS: Overall survival
OSS: Organ-sparing surgery
pN: Pathological inguinal lymph node status
RFS: Recurrence-free survival
rLAD: Radical inguinal lymphadenectomy
SCC: Squamous cell carcinoma
