# Supplementary figures and images for: Organ-sparing surgery of penile cancer: higher rate of local recurrence yet no impact on overall survival
Source: World J Urol. 2019 May 6;38(2):417–24. doi: 10.1007/s00345-019-02793-9 (PMC6994547; doi:10.1007/s00345-019-02793-9)

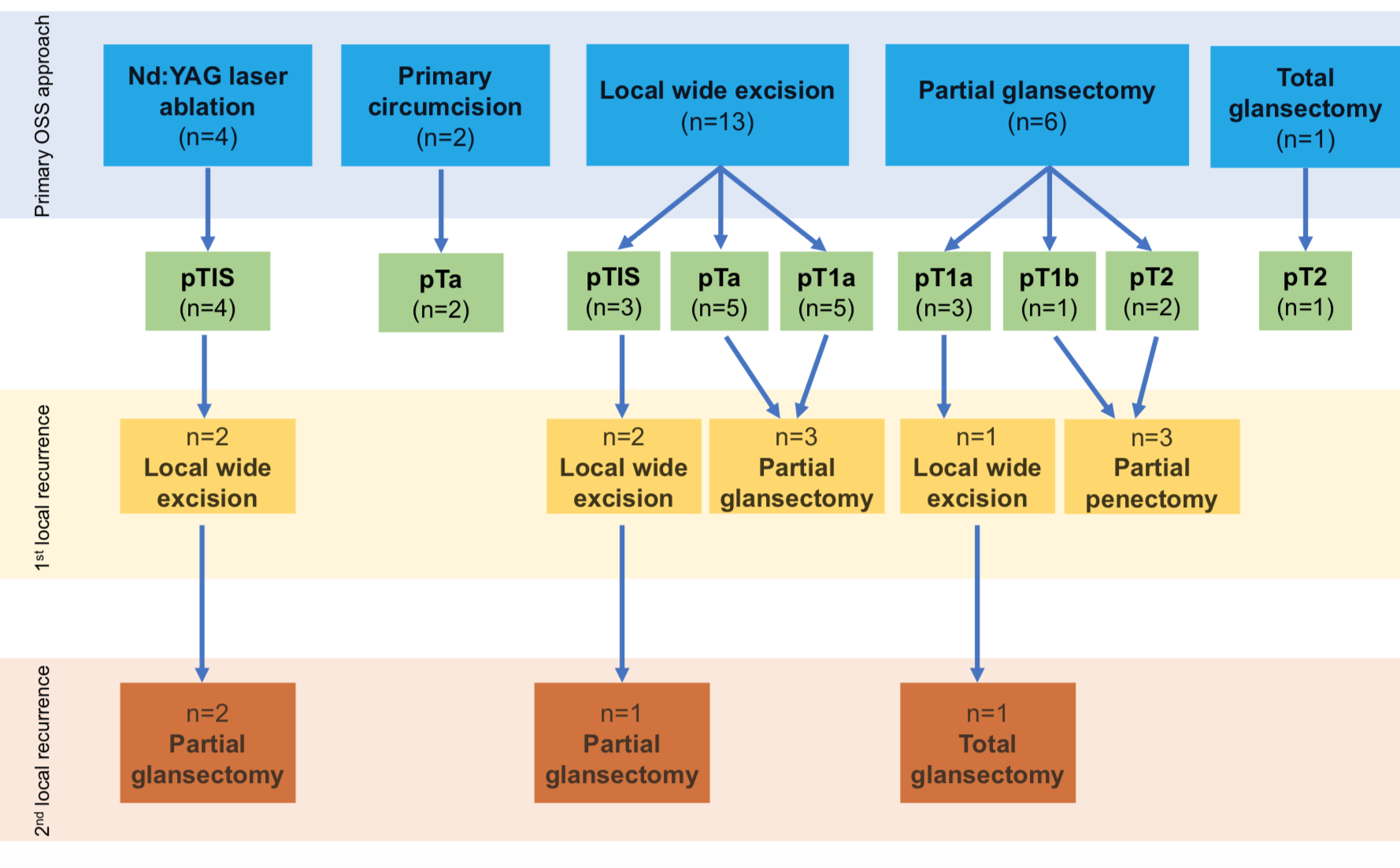

Supplement: Supplementary file 1 — Supplementary Figure 1. Schematic overview of OSS approach, pT stage and local recurrence rates (TIFF 7658 kb) [file 345_2019_2793_MOESM1_ESM.tiff]

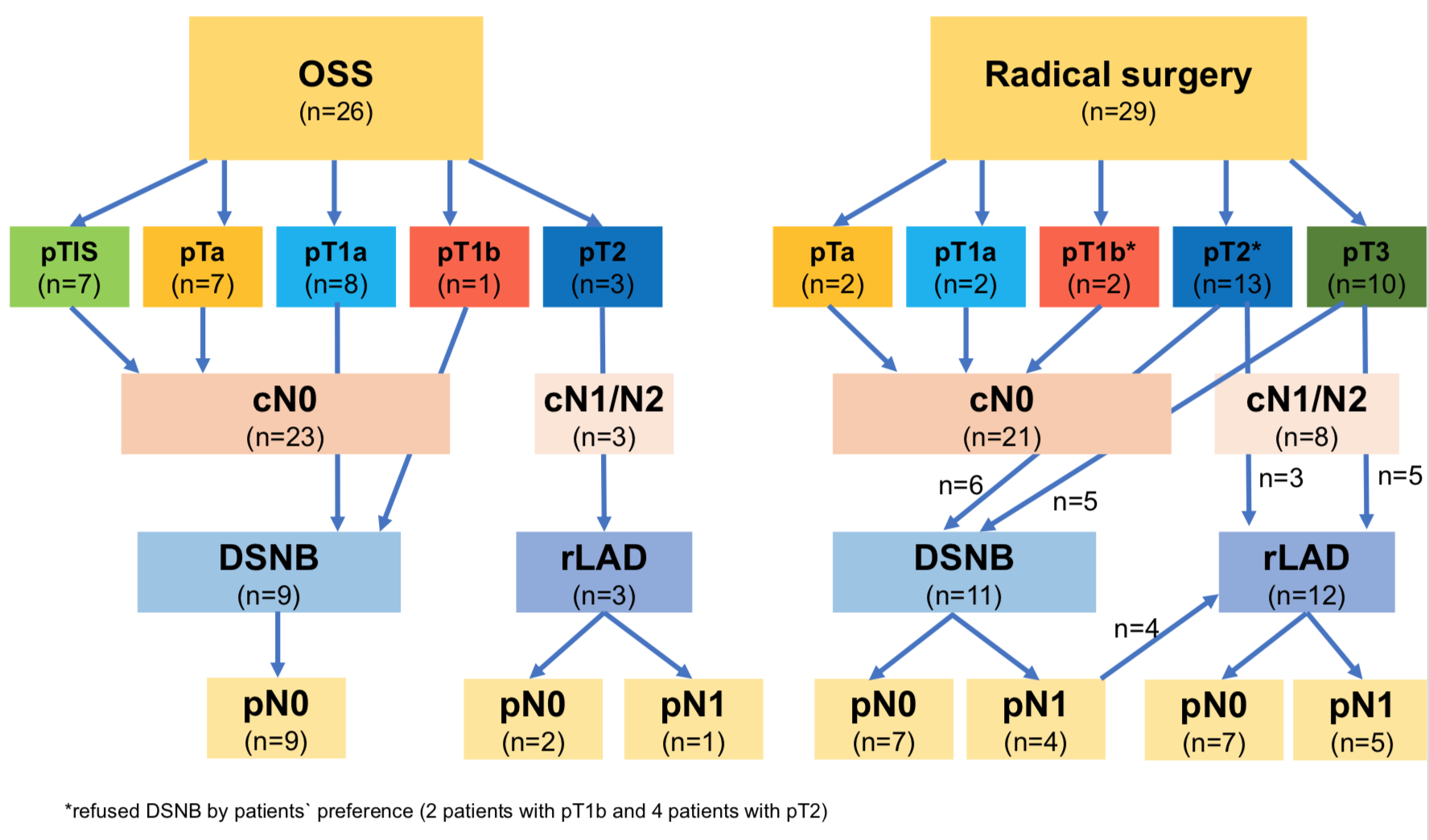

Supplement: Supplementary file 2 — Supplementary Figure 2. Schematic overview of inguinal lymph node management stratified by penile surgical approach (OSS vs. partial/total penectomy) (TIFF 7247 kb) [file 345_2019_2793_MOESM2_ESM.tiff]
